# Rapid whole-genome sequencing of *Plasmodium* DNA from cryptic malaria cases in UK travellers provides insights into infection origins, transmission and antimalarial resistance

**DOI:** 10.1093/jtm/taag028

**Published:** 2026-04-16

**Authors:** Mark K I Tan, Nina Billows, Debbie Nolder, Sophie Moss, Jody E Phelan, Joseph Thorpe, Jonathan L Edgeworth, Colin J Sutherland, Peter L Chiodini, Susana Campino, Taane G Clark

**Affiliations:** Faculty of Infectious and Tropical Diseases, London School of Hygiene and Tropical Medicine (LSHTM), Keppel Street, Camden, London WC1E 7HT, UK; Department of Infectious Diseases, King’s College London, St Thomas' Street, London SE1 9RT, UK; Centre for Clinical Infection and Diagnostics Research, Guy’s & St. Thomas’ NHS Hospital, St Thomas' Street, London SE1 9RT, UK; Faculty of Infectious and Tropical Diseases, London School of Hygiene and Tropical Medicine (LSHTM), Keppel Street, Camden, London WC1E 7HT, UK; Faculty of Infectious and Tropical Diseases, London School of Hygiene and Tropical Medicine (LSHTM), Keppel Street, Camden, London WC1E 7HT, UK; UK Health Security Agency (UKHSA) Malaria Reference Laboratory, LSHTM, Keppel Street, Camden, London WC1E 7HT, UK; Faculty of Infectious and Tropical Diseases, London School of Hygiene and Tropical Medicine (LSHTM), Keppel Street, Camden, London WC1E 7HT, UK; Faculty of Infectious and Tropical Diseases, London School of Hygiene and Tropical Medicine (LSHTM), Keppel Street, Camden, London WC1E 7HT, UK; Faculty of Infectious and Tropical Diseases, London School of Hygiene and Tropical Medicine (LSHTM), Keppel Street, Camden, London WC1E 7HT, UK; Department of Infectious Diseases, King’s College London, St Thomas' Street, London SE1 9RT, UK; Centre for Clinical Infection and Diagnostics Research, Guy’s & St. Thomas’ NHS Hospital, St Thomas' Street, London SE1 9RT, UK; Faculty of Infectious and Tropical Diseases, London School of Hygiene and Tropical Medicine (LSHTM), Keppel Street, Camden, London WC1E 7HT, UK; UK Health Security Agency (UKHSA) Malaria Reference Laboratory, LSHTM, Keppel Street, Camden, London WC1E 7HT, UK; UK Health Security Agency (UKHSA) Malaria Reference Laboratory, LSHTM, Keppel Street, Camden, London WC1E 7HT, UK; Faculty of Infectious and Tropical Diseases, London School of Hygiene and Tropical Medicine (LSHTM), Keppel Street, Camden, London WC1E 7HT, UK; Faculty of Infectious and Tropical Diseases, London School of Hygiene and Tropical Medicine (LSHTM), Keppel Street, Camden, London WC1E 7HT, UK; Faculty of Epidemiology and Population Health, LSHTM, Keppel Street, Camden, London WC1E 7HT, UK

**Keywords:** cryptic malaria, *Plasmodium* parasites, whole-genome sequencing, malaria profiling, drug resistance, transmission, geographic origins

## Abstract

**Background:**

The UK reports approximately 2000 imported malaria cases annually, necessitating effective surveillance to determine infection sources and transmission routes, inform strategies for prevention and detect molecular markers of drug resistance that may compromise treatment outcomes. Defined by their unclear route of infection, cryptic malaria cases pose a particular challenge for malaria surveillance because they may signify undetected localized transmission or malaria re-introduction and therefore necessitate additional public health resources and epidemiological investigations. Here, we demonstrate the utility of near-real-time whole-genome sequencing (WGS) in providing high genomic resolution and detailed molecular characterization to help resolve cryptic malaria cases in the UK.

**Methods:**

*Plasmodium* DNA (9 isolates) sourced from clinical blood samples underwent WGS using either Illumina or Oxford Nanopore Technologies (ONT) platforms. Sequence data were rapidly analysed with *Malaria-Profiler*, which performs read mapping, variant calling, quality control, drug resistance prediction and artificial intelligence (AI)-based geographic origin inference using a reference database of more than 15 000 isolate genomes. *Plasmodium ovale* spp. and *P. falciparum* infections identified among family members were further analysed to assess parasite relatedness using identity-by-descent and multiplicity of infection approaches to investigate transmission clusters.

**Results:**

Using a combination of Illumina and ONT WGS platforms alongside *Malaria-Profiler*, we rapidly profiled parasites from four cryptic *P. falciparum* malaria cases in the UK, identifying drug resistance markers and predicting geographic origins through AI-based methods. We also applied WGS to family-related clusters of *P. ovale* spp. and *P. falciparum* cases, confirming (sub)species identities and enabling fine-scale transmission cluster analysis.

**Conclusions:**

This study highlights the power of real-time WGS and AI-enhanced tools for high-resolution malaria genomic surveillance. By enabling rapid characterization of cryptic and imported cases, this approach supports timely public health responses, including targeted epidemiological investigations and, where appropriate, the de-escalation of entomological surveillance. In doing so, this approach helps sustain malaria elimination in non-endemic settings.

## Background

Malaria, caused by *Plasmodium* parasites, remains a significant global public health challenge, with an estimated 282 million cases and 610 000 deaths reported across 80 countries in 2024.^1^ The disease predominantly affects tropical and subtropical regions, particularly sub-Saharan Africa, where mortality is highest among children under five. Globalization, including increased international travel, trade and transport, has facilitated the importation of malaria cases to non-endemic countries, with rare instances of re-introduction to local mosquito populations in some places, including mainland Europe and the USA.^2^^,^^3^ In Europe, cases of airport and luggage malaria, caused by *Plasmodium*-infected mosquitoes transported via aircraft, have also risen.^4^^,^^5^ Approximately 2000 imported malaria cases are reported annually in the UK, and while *P. falciparum* infections account for the majority of them, *P. vivax*, *P. ovale* spp., *P. malariae* and *P. knowlesi* are also encountered.^3^ These patterns of importation highlight the need for advanced molecular tools to support cross-species malaria surveillance and case investigation in non-endemic settings, enabling improved clinical decision-making, preventive travel medicine strategies and infection control policy.

In resource-rich, non-endemic settings, malaria is typically diagnosed using microscopy, supported by lateral flow rapid diagnostic tests. However, each year in the UK, a subset of malaria cases arises without a clear history of travel to endemic regions. These are classified as cryptic in origin, prompting investigation by health protection teams to identify possible sources of infection. Malaria was historically endemic in the UK, with transmission declining steadily during the twentieth century and the last indigenous case was reported in 1957.^6^ In light of this history, ongoing global warming, and the continued presence of competent malaria vectors in the UK, such as *Anopheles atroparvus* and *An. plumbeus*,^7^ there remains a potential risk of malaria re-introduction and re-establishment. Consequently, determining the origins of cryptic malaria cases is essential to ascertain whether local mosquito transmission has occurred and to ensure that appropriate public health responses can be implemented when necessary. Recent advances in sequencing technologies, such as Illumina and Oxford Nanopore Technologies (ONT) platforms, in combination with informatics platforms,^8^ now offer rapid, high-resolution genomic profiling that can aid in resolving such cryptic cases. Whole-genome sequencing (WGS) of *Plasmodium* DNA from patient blood samples can accurately identify the infecting species and subspecies, detect mixed-species infections, characterize drug resistance markers and infer likely geographic origins. This information can help determine whether a case is imported or locally acquired, support personalized treatment strategies and identify potential transmission hotspots.^8^

The UK Health Security Agency Malaria Reference Laboratory (UKHSA-MRL), based at the London School of Hygiene & Tropical Medicine (LSHTM), receives referred blood films and whole blood samples from across England, Northern Ireland and Wales, as part of an enhanced national malaria surveillance system. The laboratory also plays a key role in investigating malaria cases of unknown origin, which are classified as cryptic. In the UK, malaria cases without a recent travel history to endemic areas are investigated by UKHSA to determine if they fall into one of several explanatory categories^9^: (i) acute infection acquired abroad but missed due to incomplete travel history; (ii) delayed detection of a previously acquired infection; (iii) importation of an infected mosquito (e.g. airport or luggage malaria, also called ‘Odyssean malaria’) or (iv) person-to-person transmission in the UK via direct contact with infected blood or tissues. However, some cases cannot be readily assigned to one of these scenarios with the information available, which is usually limited to clinical findings, travel history and a timeline of symptoms and parasite detection.

Advances in DNA sequencing, genomic analysis and artificial intelligence (AI) offer new opportunities to characterize *Plasmodium* infections with high resolution, including determining the likely geographical origin of parasites and identifying genetic markers of drug resistance.^10–12^ These tools can also assess whether multiple cases, such as those occurring within families, are genetically related, providing evidence of shared exposure routes or potential local transmission. Genome-wide similarity analyses and identity-by-descent methods can identify transmission clusters and clarify epidemiological links between cases.

The recent introduction of in-house WGS capabilities at the UKHSA-MRL has enhanced the investigation of cryptic malaria cases in the UK. Here, we demonstrate the utility of this approach through the analysis of four recent *P. falciparum* cases and a family-related cluster of three *P. ovale* spp. infections; each case illustrates the value of an integrated genomics and informatics framework in supporting public health investigations.

## Methods

### Whole genome sequencing of *Plasmodium* species

As part of UKHSA investigations, the species of malaria parasites present in samples referred from clinical malaria cases are initially confirmed using microscopy, followed by quantitative and nested PCR (qPCR, nPCR). For WGS, DNA is extracted from patient blood samples and subjected to selective whole-genome amplification (SWGA), which enriches *Plasmodium* DNA using established primer sets; this typically achieves a 10-fold increase to concentrations exceeding 100 ng/μL.^13–15^ Sequencing is performed at LSHTM using either the Illumina MiSeq or ONT MinION/PromethION platforms. The Illumina sequencing data analysis pipeline includes alignment of paired-end reads to reference genomes (Pf3D7 v3, Poc221 v1 and Pow222 v1) using BWA-MEM, followed by variant calling with GATK tools (see^15^). ONT raw reads are processed via a custom Nextflow pipeline that includes basecalling with Dorado (with --trim ON), taxonomic filtering using Kraken2 to remove non-*Plasmodium* contaminants and quality control using PycoQC. For ONT, the turnaround time from DNA extraction to sequence data is less than 8 hours, while Illumina-based sequencing takes approximately 24 hours. All sequencing and bioinformatic analyses are performed blind to the patient’s clinical presentation and travel history to reduce bias. The processing of clinical samples by the UKHSA-MRL at LSHTM for diagnostic and investigative purposes has been approved by the UK National Research Ethics Service (Ref: 18/LO/0738) and the LSHTM Research Ethics Committee (Ref: 14710).

### Integrated genomic surveillance of transmission, resistance and origin

All sequence data were rapidly analysed using the *Malaria-Profiler* tool, with complete profiling typically taking < 5 minutes.^8^ This tool integrates bioinformatic pre-processing (including read mapping, variant calling and quality control) with the rapid identification of drug resistance markers, multiplicity of infection estimation (MOI) and geographic origin prediction. It leverages a curated database of over 15 000 *Plasmodium* with WGS data and employs AI-driven models based on barcode markers for geographic assignment.^8^^,^^11^ For Illumina data, raw reads are trimmed using *Trimmomatic* software (v0.39),^16^ then aligned with *BWA* (v0.7.18-r1243-dirty),^17^ while ONT data are aligned using *Minimap2* (v2.1.1-r341).^18^ Variant calling is performed using *GATK* (v4.1.4.1) for Illumina reads and *FreeBayes* (v1.3.10) for ONT reads.^19^^,^^20^ High-quality variants (filtered by PASS and minimum depth > 5) are selected using *bcftools* (v1.17),^21^ before being analysed for known drug-resistance mutations and geographic origin. Specifically, resistance to antimalarial drugs, including chloroquine, sulfadoxine-pyrimethamine (SP) and artemisinin-based combination therapies, was evaluated using established resistance-associated markers in key parasite loci (e.g. *pfcrt*, *pfmdr1*, *pfdhfr-ts*, *pppk-dhps*, *pfkelch13*).^6^

In addition, newly sequenced *P. ovale* spp. samples (*n* = 3) underwent further downstream analysis to investigate potential transmission clustering, and were compared to public sequences (*n* = 101).^15^^,^^22^ All samples were processed using an established variant calling pipeline (https://github.com/LSHTMPathogenSeqLab/fastq2matrix), which employs *GATK* (v4.1.4.1) HaplotypeCaller to generate GVCFs, subsequently merged with a reference *P. ovale* spp. dataset. Variant quality was refined using *GATK* VariantFiltration (default parameters), and high-confidence variants were retained with *bcftools* (v1.17; minimum depth = 5). The primary objectives were to determine the *P. ovale* spp. [−*curtisi* (Poc) or -*wallikeri* (Pow)] and to assess whether transmission had occurred between family members by comparing the genomic similarity of the isolates. We aimed to distinguish between infections acquired simultaneously and potential secondary (intra-household) transmission. This was addressed through an identity-by-descent (IBD) analysis, which estimates the proportion of the genome shared among individuals due to recent common ancestry. IBD analysis was conducted in R using the *isoRelate* package (v0.1.0; https://github.com/bahlolab/isoRelate), applying a minor allele frequency threshold of 0.001 and default missingness settings. MOI was estimated by the F_WS_ statistic using the *moimix* package (https://github.com/bahlolab/moimix), which also generated the PED and MAP files required for *isoRelate* input. This pipeline was also applied to two family-related cases of *P. falciparum* infection.

## Results

Using four UK cryptic *P. falciparum* investigations (indicated by sample numbers: S399, S417, S426/S427, S428/S429) and three *P. ovale* spp. malaria cases (S438-S440) diagnosed in 2024, we demonstrate the potential to support genomic surveillance, enable the tracing of infections to their geographical origin and provide evidence of transmission (Table 1).

**Table 1:** Clinical samples and genomic profiles.

ID	Description	Predicted species	Drug resistance	Predicted [Recorded] geographical source	Likely scenario^a^
S399	Returned from East Africa, and thought to be a location non-endemic for malaria	Pf	Chloroquine, pyrimethamine	East Africa [East African island]	Imported malaria
S417	Travelled within Europe and contracted malaria	Pf	Chloroquine, sulfadoxine	West Africa [Europe]	Airport malaria
S426, S427	Resides within the UK and had a family visit from West Africa	Pf	Chloroquine, SP	West Africa [West Africa]	Imported mosquito infection
S428, S429	Mother and child holiday in Mexico, with family ties to West Africa	Pf	SP	Central-Western Africa [Mexico]	Imported mosquito
S438, S439, S440	Family of three travel to Turkey on holiday, which has cases of *P. ovale* spp.	Poc		African Continent [Turkey]	Imported malaria

Pf, *P. falciparum*; SP, sulfadoxine pyrimethamine; Poc, *P. ovale curtisi*; ACT, artemisinin-based combination therapy.

a *Airport malaria* refers to malaria acquired at or near an airport, typically through the bite of an infected mosquito that has been transported by aircraft from a malaria-endemic region; *imported mosquito infection* refers to malaria transmitted by an infected mosquito that has been transported from an endemic area, but where the actual site of transmission occurs away from the airport.

**Figure 1:**
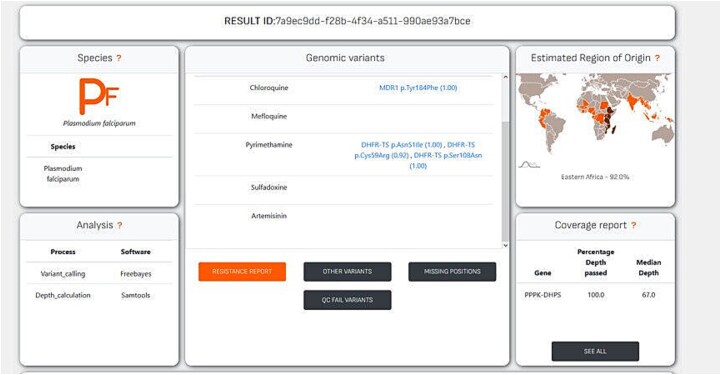
*Malaria-Profiler* prediction for cryptic *P. falciparum* sample. The web-based *Malaria-Profiler* output for S399 is shown. The screenshot provides a high-level overview of the drug-resistant mutations identified in the sample, along with a predicted geographic origin in East Africa. The recorded source—a coastal island near East Africa that was not represented in the AI training dataset—aligns with this prediction.

**S399:** An adult patient returned to the UK from travel to an undisclosed location in East Africa, and after 2 days, became unwell, being diagnosed with *P. falciparum* malaria. Amplified DNA was sequenced on the ONT platform, leading to 21 M reads (~500 bp) and a > 50-fold genome-wide coverage. *Malaria-Profiler* software revealed 464 mutations in drug-resistant candidate regions, including established variants linked to chloroquine (*pfmdr1* Tyr184Phe) and pyrimethamine (*pfdhfr-ts* Asn51Ile, Cys59Arg and Ser108Asn) resistance, but not to sulfadoxine or artemisinin-based combination therapy (ACT) (Figure 1). *Malaria-Profiler* also predicted an MOI of 1. The patient responded well to ACT treatment. The analysis indicated that the local source of infection was likely East Africa with a probability of 0.92 (Figure 1). Several potential countries of origin were identified, though none conclusively; the top three were Tanzania with a 0.25 probability, Malawi with 0.21 and Kenya with 0.18, based on database enrichment. Subsequent investigations revealed the patient had travelled to and had family ties to a coastal island situated near Eastern Africa that is regarded as malaria-free. Due to the island’s status, multiple transmission pathways may be possible, including the introduction of an infected mosquito or transmission from a recently established local focus of malaria. Moreover, the geographic prediction was shown to correlate with the patient’s travel history. The patient made a full recovery within a few days of treatment.

**S417:** An adult patient with no previous history of malaria infection had travelled within Europe by air and presented with a case of *P. falciparum* malaria. Given the absence of explanatory travel history, geographic origin was investigated by the generation of Illumina sequence data. A total of 93.2% genome coverage was achieved at > 5-fold coverage. The *Malaria-Profiler* tool identified 10 drug-resistance variants, conferring resistance to chloroquine (*pfcrt* Lys76Thr, Ala220Ser, Gln271Glu, Ile356Thr and Arg371Ile) and sulfadoxine (*pppk-dhps* Ile431Val, Ser436Ala, Ala437Gly, Ala581Gly and Ala613Ser). The sample was predicted to have a single clone (MOI = 1). The regional source of infection was identified as West Africa with 0.99 probability. Gambia (0.30 probability) and Mali (0.26 probability) were potential countries of origin. Two neighbouring countries, Guinea and Senegal, also had probabilities > 0.1. Due to the absence of any recent travel to an endemic country, the case was classified as likely airport malaria, acquired by the bite of an imported mosquito in one of the European cities visited.

**S426/S427:** An adolescent patient residing in the UK presented with *P. falciparum* malaria despite no recent travel to a malaria-endemic country. DNA extracted from two whole blood samples from the patient was sequenced on the Illumina platform, achieving genome coverages of 97.7% and 98.7%, respectively, at > 5-fold depth. Samples had an estimated MOI of 1. Analysis using the *Malaria-Profiler* tool identified consistent drug-resistance profiles across both datasets. Mutations were detected in multiple loci associated with resistance to chloroquine, sulfadoxine and pyrimethamine, including six mutations in *pfcrt* (Met74Ile, Asn75Glu, Lys76Thr, Ala220Ser, Gln271Glu, Ile356Thr, Arg371Ile), one mutation in *pfmdr1* (Tyr184Phe), one in *pppk-dhps* (Ala437Gly) and three in *pfdhfr-ts* (Asn51Ile, Cys59Arg, Ser108Asn). The patient recovered following treatment. Geographic source prediction using AI-based modelling assigned West Africa as the most likely origin with a probability of 0.99 for both samples. Country-level predictions suggested Gambia (44%) and Mali (>10%) as the most probable sources. Although a relative was reported to have recently returned from West Africa, the patient themselves had not travelled abroad. The malaria case was therefore classified as ‘Odyssean malaria’, likely acquired from an infected mosquito inadvertently imported via luggage or cargo arriving through the local maritime docks.

**S428/S429.** A patient (S428) under the age of 5 years and their mother (S429) presented with *P. falciparum* malaria following a holiday in Mexico. DNA extracted from two whole blood samples was sequenced on an Illumina platform. The resulting genome coverages were 97.7% (S428) and 84.2% (S429) at > 5-fold depth. Both samples contained a single clone only (MOI = 1). Analysis revealed multiple drug-resistance mutations associated with antifolate resistance in both samples. Specifically, three mutations in the *pfdhfr-ts* gene were detected: Asn51Ile, Cys59Arg and Ser108Asn. Additionally, five mutations were identified in the *pppk-dhps* gene: Ile431Val, Ser436Ala, Ala437Gly, Ala581Gly and Ala613Ser, suggesting resistance to SP in both samples. The predicted regional source of infection did not align with the patient’s recent travel history. AI-based geographic analysis indicated the highest likelihoods for Central Africa (57–75%) and West Africa (23–42%) as the source regions. At the country level, Cameroon and Nigeria were the most probable origins, with predictive probabilities of 0.50 and 0.69, respectively. The mother was from West Africa, but she had not travelled outside the UK for 5 years. Although the patients had recently travelled to Mexico, the genomic findings, combined with epidemiological context, supported a diagnosis of baggage malaria—likely resulting from exposure to an infected mosquito inadvertently transported by a family member returning from West Africa. This was further supported by IBD-relatedness analysis, whereby parasite isolates S428 and S429 were identical by descent over 99.5% of the entire genome (across 12 379 bi-allelic SNPs). Both the mother and child made a full recovery.

**Figure 2:**
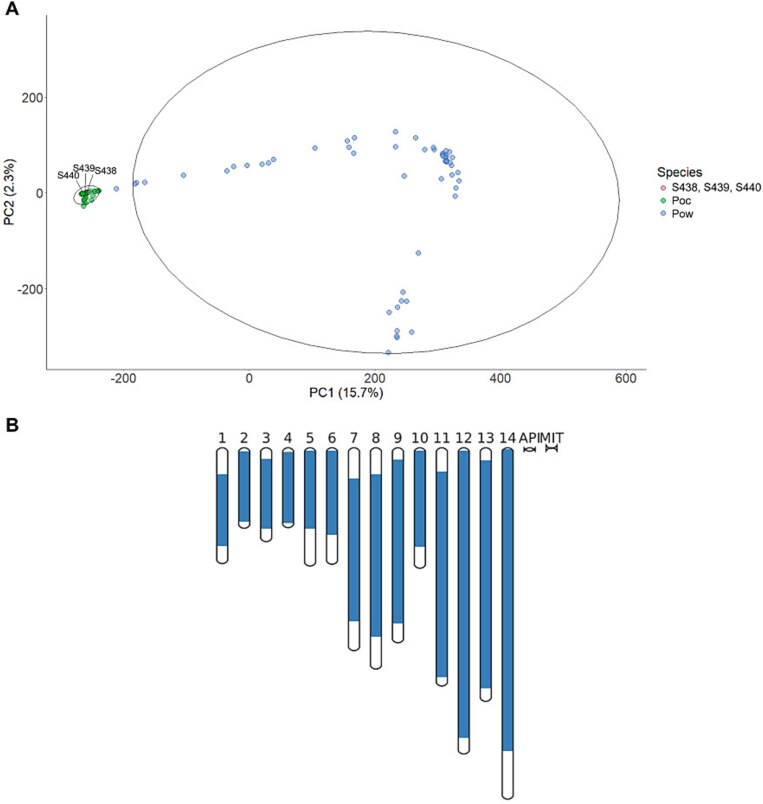
Cryptic *Plasmodium ovale* spp. samples cluster with known species and share highly identical genomic regions. (A) Principal component (PC) analysis plot of *P. ovale* spp. isolates, including *P. ovale curtisi* (Poc) (*n* = 40, green points), *P. ovale wallikeri* (Pow) (*n* = 55, blue points) and the cryptic samples (*n* = 3). The cryptic samples cluster with the *P. ovale curtisi* species, indicating a closer genetic relationship. (B) Across the 14 chromosomes, mitochondria (MIT) and apicoplast (API) genomes, genomic segments shared among the three cryptic *P. ovale curtisi* samples, showing regions with an identity-by-descent (IBD) proportion greater than 0.99 (blue), reflecting that the three samples are part of the same transmission chain. For chromosomes 1–14, sub-telomeric regions are highly variable, leading to IBD < 0.99 (white).

**S438-S440:** Three family members, a child (S440), an adult female (S438) and an adult male (S439), presented with *P. ovale* spp. malaria in the UK following recent travel to Turkey. Notably, the child and the female adult developed symptoms ~ 2 weeks after the adult male. WGS was performed on all three cases using the Illumina MiSeq platform, achieving moderate to high genome coverage (48–96%) at ≥ 5-fold depth. To investigate species identity and relatedness, the cryptic samples were compared to 101 publicly available *P. ovale* spp. genomes.^15^^,^^22^ Population structure analysis revealed that all three clustered with *P. ovale curtisi* (Figure 2A). However, due to limited geographical diversity in the reference dataset, comprising mostly African isolates, precise geographic attribution was not possible. Nonetheless, the cryptic samples grouped with other *P. ovale curtisi* genomes from multiple African countries (data not shown). Furthermore, to assess genetic relatedness, we examined IBD across the genome, identifying clusters of samples sharing > 95% and > 99% IBD (Figure 2B). At the more stringent 99% threshold, two distinct clusters were observed: one consisting of two public genomes from Sudan, and the other comprising all three cryptic samples. The cryptic samples also showed elevated pairwise IBD fractions relative to those typically observed within the same region or household (median IBD < 0.01), indicating strong genetic relatedness. This was further supported by low pairwise SNP distances among the cryptic samples (mean = 2268 SNPs), compared to the broader *P. ovale curtisi* population (mean = 15 062 SNPs). Collectively, these findings strongly suggest that the three *P. ovale curtisi* infections were genetically closely related and likely derived from a common source. Despite staggered symptom onset, the high degree of genetic similarity indicates that transmission most likely occurred at the same time and/or location, potentially through a shared exposure event, such as within or close to a household setting. All three patients recovered well after treatment.

## Discussion

These cases demonstrate the potential of integrating WGS with AI-driven analysis to enable personalized treatment and strengthen global malaria surveillance. While challenges remain, such as the need for large-scale studies to evaluate cost-effectiveness, our findings demonstrate that parasite genomic data can be generated and analysed within 24 hours, facilitating improved patient outcomes through personalized medicine. Recent advances, including rapid barcoding during pre-sequencing sample preparation, targeted amplicon sequencing for higher throughput and real-time monitoring of sequencing progress to optimise run times, collectively reduce costs and turnaround times. Consistent with expectations, Illumina and ONT sequencing results showed high concordance; however, ONT offers distinct advantages through longer read lengths and platform portability, enabling applications in low-resource and field settings.

Expanding the diversity and geographic representation of *Plasmodium* genome databases, especially through ongoing sequencing of UKHSA isolates from travellers to underrepresented geographic regions, will enhance the resolution and accuracy of AI models. Further refinement of these models via more sophisticated machine learning approaches will increase their utility in clinical and public health contexts.^8^^,^^11^ Identifying the origins of cryptic malaria cases is critical for surveillance, enabling timely notification of institutions in non-malaria endemic regions about potential novel transmission events. Furthermore, the detection of emerging drug resistance in a broadly based sentinel population not at risk of reinfection, in whom genotype can be matched to phenotype and clinical outcome, is of both public health and clinical importance. More generally, the discriminatory power of the implemented pipeline can support investigations of cryptic malaria cases and guide the targeted deployment of resources across clinical, epidemiological and entomological activities, including surveillance. Such surveillance may also detect resurgence in areas previously considered malaria-free.^23^ Amid global elimination efforts, the increasing reports of artemisinin partial resistance across East Africa underscore the urgent need for robust drug resistance monitoring matched to likely parasite origins.^24–26^ An integrated genomics and informatics framework is well-positioned to meet this need.

Importantly, the rapid turnaround of genomic data enables real-time clinical decision support. Unlike some clinically relevant bacterial pathogens, malaria parasites require prolonged culture periods to assess drug sensitivity, which often delays actionable results beyond the clinical window. Moreover, continuous *in vitro* culture is currently feasible only for *P. falciparum* and *P. knowlesi*. Given growing concerns about artemisinin-based combination therapy failures in *P. falciparum*, earlier detection of resistance markers is critical to inform effective treatment strategies. Our work also demonstrates the value of WGS for identifying transmission clusters, aiding in distinguishing between *de novo* emergence, importation and onward transmission. Assisting in the detection of these transmission clusters, IBD analysis was crucial for identifying the relatedness between familial cases, thereby reducing the likelihood of independent transmission events. More broadly, IBD analysis can provide evidence of molecular epidemiological links between cases. For example, in hospital settings where a secondary infection occurs while a malaria patient is being treated nearby, rapid genomic assessment can help determine whether transmission occurred directly via blood exposure or through a local mosquito vector. Such genomic analyses are valuable for attaining and sustaining malaria elimination by translating *Plasmodium* sequence data into actionable public health responses. For instance, new national strategies in Senegal were developed to mitigate malaria infection risks during a pilgrimage after cases along the route were identified as genetically related through IBD analysis.^27^ Similarly, the cryptic malaria cases resolved here further demonstrate the practical value of an integrated genomic–informatics workflow.

Utilizing near-real-time sequencing on both ONT and Illumina platforms, combined with the *Malaria-Profiler* tool, we successfully predicted the geographic origins and drug resistance profiles of *Plasmodium* infections. This AI-enabled approach also illuminated transmission dynamics across species, including *P. ovale* spp., revealing clusters that enhance understanding of local and international spread. Collectively, these methods establish a robust framework for real-time genomic surveillance, central to understanding cryptic malaria cases in the UK and poised for continuous improvement as genomic data from a broader geographic spectrum accumulate.
